# The landscape of evolution

**DOI:** 10.1093/nsr/nwag211

**Published:** 2026-04-06

**Authors:** Mihai N Ducea

**Affiliations:** Faculty of Geology and Geophysics, University of Bucharest, Romania; Department of Geosciences, University of Arizona, USA

Tectonic processes are frequently cited as drivers of biological change, but the precise mechanisms by which they shape terrestrial ecosystems often remain elusive. A new study by Wang *et al.* [1] bridges this gap by linking the dramatic flourishing of the renowned Yanliao and Jehol biotas to the tectonic transformation of Northeast Asia during the Jurassic and Early Cretaceous. By reconstructing time-integrated paleoelevations and regional temperatures, researchers have revealed how the rise of a ‘Mesozoic Tibet’—the East Asian Highland—created the complex, vertically zoned environments necessary to foster exceptional biodiversity.

Using chemical paleo-mohometry [2], a multi-proxy approach estimating crustal thicknesses and surface elevations in ancient orogens from igneous rock compositions, completed with previously published clumped isotopes and basalt vesicularity data, the authors estimate that Mid–Late Jurassic plate convergence pushed regional elevations to between 3.0 and 4.4 km [1].

This massive highland acted as a ‘sky island’ archipelago, where high relief and complex topography generated a spectrum of ecological niches:

• The Yanliao Biota (∼167–156 Ma): the study suggests that this high-relief landscape provided relatively cool refugia within a globally warm climate, facilitating the radiation of early mammals and the transition from dinosaurs to birds.

• The Jehol Biota (∼135–119 Ma): following a period of aridification and ecological disruption, Early Cretaceous tectonic extension further ‘sculpted’ the landscape. The increased topographic ruggedness and the formation of numerous rift basins expanded available surface area and habitat connectivity, triggering the emergence of the diverse Jehol lineages, including flowering plants and eutherian mammals.

These findings highlight that the East Asian Highland served as a critical biodiversity cradle. The region’s topographic complexity offered species the opportunity to track suitable climates and withstand global oscillations, significantly improving the resilience of local ecosystems. Interestingly, the period from the Middle Jurassic through the Early Cretaceous was characterized by relatively high global temperatures [3]. Despite this prolonged warmth, the East Asian Highland provided relatively cool and stable refugia, allowing these biotas to thrive even as global temperatures fluctuated.

More broadly, this research demonstrates that by controlling magmatism, crustal thickness, and basin formation, plate boundary dynamics indirectly but profoundly steer the path of terrestrial evolution. It also highlights the growing potential of multi-proxy chemical mohometry, particularly when integrated with expanding regional or global magmatic datasets, to reconstruct the paleogeography of ancient eroded orogens commonly missing records of (near-)surface processes. Such approaches offer new opportunities to link tectonics, surface processes, and biodiversity within an Earth system science framework [4].
